# Analysis of Movement and Activities of Handball Players Using Deep Neural Networks

**DOI:** 10.3390/jimaging9040080

**Published:** 2023-04-13

**Authors:** Kristina Host, Miran Pobar, Marina Ivasic-Kos

**Affiliations:** 1 Faculty of Informatics and Digital Technologies, University of Rijeka, 51000 Rijeka, Croatia; 2 Centre for Artificial Intelligence and Cybersecurity, University of Rijeka, 51000 Rijeka, Croatia

**Keywords:** sports, object detector, object tracking, action recognition, video analysis, YOLO, Mask R-CNN, DeepSORT, BoT SORT, I3D

## Abstract

This paper focuses on image and video content analysis of handball scenes and applying deep learning methods for detecting and tracking the players and recognizing their activities. Handball is a team sport of two teams played indoors with the ball with well-defined goals and rules. The game is dynamic, with fourteen players moving quickly throughout the field in different directions, changing positions and roles from defensive to offensive, and performing different techniques and actions. Such dynamic team sports present challenging and demanding scenarios for both the object detector and the tracking algorithms and other computer vision tasks, such as action recognition and localization, with much room for improvement of existing algorithms. The aim of the paper is to explore the computer vision-based solutions for recognizing player actions that can be applied in unconstrained handball scenes with no additional sensors and with modest requirements, allowing a broader adoption of computer vision applications in both professional and amateur settings. This paper presents semi-manual creation of custom handball action dataset based on automatic player detection and tracking, and models for handball action recognition and localization using Inflated 3D Networks (I3D). For the task of player and ball detection, different configurations of You Only Look Once (YOLO) and Mask Region-Based Convolutional Neural Network (Mask R-CNN) models fine-tuned on custom handball datasets are compared to original YOLOv7 model to select the best detector that will be used for tracking-by-detection algorithms. For the player tracking, DeepSORT and Bag of tricks for SORT (BoT SORT) algorithms with Mask R-CNN and YOLO detectors were tested and compared. For the task of action recognition, I3D multi-class model and ensemble of binary I3D models are trained with different input frame lengths and frame selection strategies, and the best solution is proposed for handball action recognition. The obtained action recognition models perform well on the test set with nine handball action classes, with average F1 measures of 0.69 and 0.75 for ensemble and multi-class classifiers, respectively. They can be used to index handball videos to facilitate retrieval automatically. Finally, some open issues, challenges in applying deep learning methods in such a dynamic sports environment, and direction for future development will be discussed.

## 1. Introduction

The sports domain offers many opportunities to apply computer vision (CV) to help athletes, coaches, and teams reach their goals by enabling more efficient and detailed analysis and tracking of performance, or for the broadcasters to improve the experience for the viewers—however, the fast pace, complex dynamics, and interactions in many sports challenges implementing CV solutions. Additionally, achieving the high reliability required in commercial applications often leads to expensive solutions involving many specialized high-speed cameras, sensors, and support staff that are sustainable only at the top competitive level of the most popular sports, such as football, baseball, or basketball. Thus, it is of great research interest to develop more advanced and robust CV-based solutions with fewer technical constraints and requirements that would allow a much broader adoption of CV applications in both professional and amateur sports.

In the research, there is a great focus on sports, such as basketball, soccer, and volleyball. For example, in [1], the authors applied real-time detection with the second version of You Only Look Once (YOLO) [2] algorithm to detect players, then the Simple Real time Tracker (SORT) algorithm [3] to track them, and finally, an Long short-term memory (LSTM) for action recognition on basketball videos from the National Collegiate Athletic Association (NCAA) Basketball Dataset [4]. In [5], the author created a custom dataset with 6 basketball actions in 37 annotated videos for pose-based action recognition involving multiple players. The author combined pose estimation with SORT tracking to obtain key point vectors, then used them to classify the actions using Multilayer Perceptron (MLP), LSTM, and Bidirectional Long Short-Term Memory (BiLSTM) networks. To summarize long soccer videos, the authors [6] pick relevant segments to include by temporally localizing five actions of interest using an LSTM-based network on top of a 3D Convolutional Neural Network (3D-CNN) feature extractor based on ResNet trained on a custom soccer dataset. Finally, in [7], the authors focused on a fine-grained action recognition problem of distinguishing between successful or failed ball-stopping action in soccer training. The authors first used a YoloV3-based model for player and ball detection. If only one player and the ball exist in the frame, they used the detected bounding boxes to create trajectories that were then classified using an LSTM-based network. The networks were trained on a custom dataset of 2543 annotated ball-stopping action videos from training sessions.

Researchers have recently studied group activity in team sports and individual actions. For example, in [8], the authors used a two-stream 3D-CNN model with motion patterns and visual key information to recognize group activities in basketball and predict whether there will be a score. In [9], the authors designed an LSTM model for recognizing individual action dynamics to steer another LSTM model to recognize group activities in volleyball. They evaluated their model on existing and custom volleyball datasets with YouTube videos. In [10], the authors compare and evaluate the performance of handball players while performing “Throw” action. They used dynamic time wrapping to compare skeleton data gathered from Red Green Blue-Depth (RGB-D) images, focusing on the central angles responsible for the action. In [11], the authors considered recognizing three group activities in soccer with different self-attention models, such as the transformer and vision-based models with Inflated 3D Networks( I3D) as a backbone. In [12], the focus is on recognition of six team activities, such as basic defense or offense fast break from a top-down view using new group activity features computed from player positions.

There are many publicly available datasets that contain data specific to the sports domain. Some examples of publicly available datasets are the UCF Sports Action Data Set [13], Olympic Sports Dataset [14], the Sports-1M dataset [15], and the Sports Videos in The Wild (SVW) dataset [16]. However, although there are publicly available datasets for the sports domain, they often do not meet the specific requirements for which a model needs to be built, thus, many authors create their own custom datasets that are tailored to model building for their specific task, despite the effort and cost involved in data collection, labeling and preparation. Two datasets that focus on fine-grained actions are the FineGym dataset [17] and the MultiSport dataset [18].

A more extensive overview of related work in action recognition in sports is given in [19]. In the review, it was noted that there is a significant increase in interest in actions recognition in ball sports, but also that all sports, even Olympic ones, are not equally represented in the research. One of the Olympic sports that is particularly popular in Europe and is underrepresented in research is handball.

Therefore, the subject of our research in this paper is focused on the application of deep learning methods and building computer vision models for handball. The goal of this work is to train models for automatic player detection and player tracking, and recognition of the actions they perform and their temporal localization on handball videos recorded with only one camera, from a position accessible to spectators and coaches. The key issue is to make models that can be easily used by everyone, especially recreationists, young athletes, and athletes in small clubs, and everyone else interested in sports without expensive equipment and cameras to track their performance and improve the usability of recorded videos in the case of handball.

This work is a continuation of our comprehensive research project in which we dealt with player and ball detection [20], tracking the player in video [21], and determining the key player in the field [22]. We used player detection and tracking models to create an action recognition dataset in a way that shortens the time-consuming and tedious work of tagging video sequences. The paper briefly describes this procedure for the semi-manual creation of a dataset for training an action recognition model based on expert evaluation of the results obtained by automatic player detection and tracking. The paper also presents the new UNIRI-HBD_v2 dataset that was created in this way and was used to train the models for handball actions recognition.

The contributions of this paper include the analysis of two different strategies for training the action recognition models, namely, a comparison of a single multi-class model capable of assigning one action label to each sequence and of an ensemble of binary classifiers where each classifier can assign or not assign its corresponding label, resulting in multi-label classification.

Handball actions vary widely in duration, from 10 to 80 frames, and have important segments in different parts of the video sequence. Since action recognition models require a fixed number of input frames, the influence of the length of the input sequence was separately analyzed on the performance of the classification of isolated actions and on the temporal localization of actions in unconstrained videos. In the case of temporal action localization, the considered time window may include only a part of the action or contain parts of different actions. All experiments were conducted on a custom dataset of annotated handball videos [23].

The next section presents materials and methods used. The first subsection presents the most important methods used for semi-manual action dataset creation to accomplish the task of action recognition in handball, including object detection and person tracking. The second subsection explains action recognition and temporal localization tasks, followed by a description of the algorithms used in the experiments. The third section presents the action recognition and temporal localization experiments and fourth obtained results, followed by a discussion. Finally, the last section gives a conclusion with future research directions.

## 2. Materials and Methods 

### 2.1. Semi-Manual Action Dataset Creation

To train the model for different computer vision tasks on handball scenes, it was necessary to create a custom dataset since there was no suitable dataset that could be used to train the model in the handball domain. Given that the creation of a set for supervised training is a long-term and expensive process, the goal was to apply an approach that facilitates and speeds up the creation of a set.

**Figure 1:**
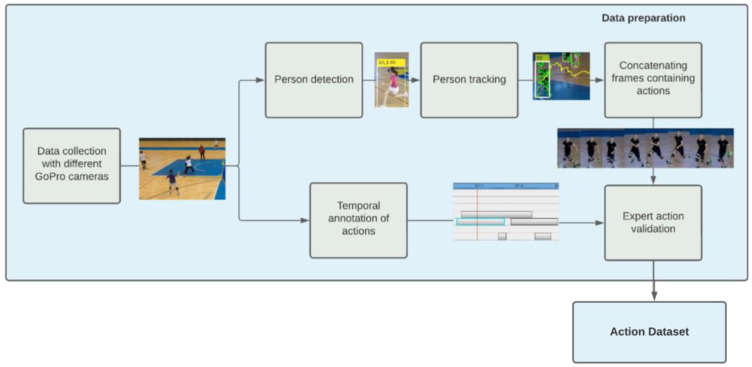
The semi-manual semi-supervised action dataset creation: the annotation of the data set includes manual temporal labeling followed by automatic spatial localization of players by means of automatic player detection and tracking, and finally, manual verification of generated action proposals.

The challenge in video tagging of handball scenes recorded during training and matches was that there are multiple players on the field at the same time performing different actions, moving in different directions, at different distances from the camera, and dynamically entering and exiting the camera’s field of view. The idea was to tag actions in videos using a person detector that automatically detects special location of players and tracking methods that follow their movements to help automatically recognize their actions. The annotation of the data set includes also manual temporal labeling where time sequences corresponding to a handball action are marked. As different actions can take place at the same time, multiple actions can be marked at some point in time, where there is no clear connection between the annotation and the spatial location where the action occurs. Figure 1 shows the basic steps of semi-manual creation of an image dataset for recognition of handball actions in scenes where multiple players are present.

Player detection and tracking isolate ongoing candidate actions from a video. The actions are performed by individual players and lastly, for a sequence of consecutive frames. At the same time, the basic handball actions, such as “Shot”, “Jump Shot”, “Running”, “Dribbling”, etc., were temporally annotated using a video annotation tool. The next step merges the tracked player with the action taking place in that time and involves manual validation of the matches to build the final ground truth data for action recognition models. Expert-assessed matching of automatically selected video clips with the performed action is crucial at this stage because the athlete tracking model still does not have satisfactory accuracy, and the goal is to populate the dataset only with accurate data that will be useful for training the model.

**Figure 2:**
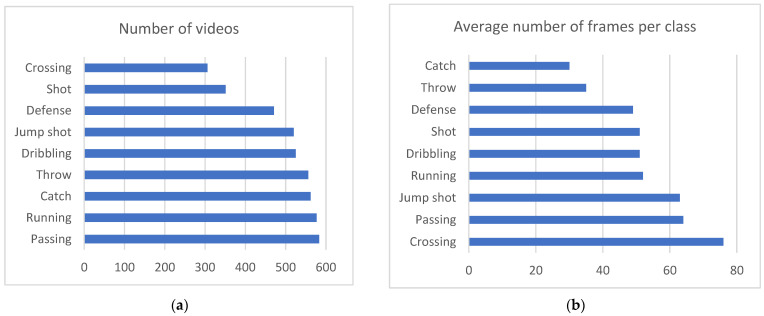
(**a**) Distribution of videos through classes, (**b**) Average number of frames in a sequence, per class.

The resulting UNIRI-HBD_v2 dataset contains 4451 sequences in 9 action classes and a “Background” class with class distribution and average length, as shown in Figure 2.

The described process of dataset creation is relatively complex, but it is important that once defined, the player detection and tracking model can be used multiple times to automatically supplement the dataset of handball actions.

#### 2.1.1. Player and Ball Detection

To recognize player actions and activities later, a handball player should be detected first, i.e., the player must be located on an image or consecutive video frame. This is a typical object detection task, where the goal is to locate all objects of a certain class (in this case, a handball player) in the image and return the spatial location and spatial extents of the detected objects, usually represented with rectangular regions.

During the preparation of the handball dataset, different configurations of Mask R-CNN [24], YOLOv3 [25], and YOLOv7 [26] models were considered for the detection of handball players and sports balls. YOLOv3 was tested in its default configuration with the pre-trained model and in custom Yolov3-PB configuration with increased input image size and fine-tuned on our UNIRI_HBD dataset of 855 images containing different sports balls and from different sources [20]. Mask R-CNN was tested in its Detectron2 implementation [27], using the model pre-trained on the COCO dataset, with the ResNeXt-101-32 × 8d and FPN backbone with standard convolutional and fully connected heads for box prediction. Here, we also present that publicly available models, such as YOLOv7 trained on large datasets, such as COCO [28], for detection of broad and general objects, can be used relatively successfully for player detection.

#### 2.1.2. Player Tracking

Tracking handball players is a multi-object tracking problem because there can be 14 players simultaneously if a handball competition is analyzed, and even more in a training session. With an object detector, all the players’ locations would be known in the ideal case. However, there is still no correspondence between the detected player bounding boxes and the players’ identities. This problem can be solved using a tracking algorithm, or tracking-by-detection algorithm, that should assign a unique ID to each player that appears in a frame and maintains that ID while that player is in the scene. To accomplish that, a tracker can use the information obtained by the object detector, such as the dimensions and the location of the bounding boxes, along with their relative positions considering previous frames with the assumption that the objects should be near their last position or move according to a known movement model, e.g., in a linear fashion. Trackers may also use visual features extracted from within the bounding boxes if the overall appearance of the tracked object maintains some constant features that can be tracked even with changing pose or orientation. Cost functions are defined using both sources of information that serve as a quantitative measure of how different a bounding box is from the presumed position or appearance of the tracked object in the next frame. The Hungarian algorithm is used to find the globally optimal assignment of identification tracks to the bounding boxes of the players obtained by the object detector so that the cost function for the assignment reaches the global minimum.

Tracking of players can be very demanding because players move quickly through the field and often change directions, challenging the assumption that the detected bounding box of a player in the next frame will be near or nearest to the last one. In addition, especially in the competition setting, the players wear similar clothes, often in colors that also appear in the “Background” (e.g., blue shirts on the blue court), further complicating tracking of appearance features that must be specifically sensitive to player identity unrelated to clothes.

In this work, we expand on the work of [29], where DeepSORT [30] was used for player tracking in conjunction with the YoloV3 [25] player detector, and additionally considered BoT-SORT [31] for tracking and Mask-RCNN and YoloV7 player detection. Deep SORT and BoT-SORT are tracking-by-detection algorithms that rely on external object detectors to detect the objects (players) on consecutive frames and use the Hungarian algorithm to solve the global assignment problem to join the player on the current frame to his tracklet from the previous frames. DeepSORT uses an appearance model to keep an inventory of visual features from previously assigned player bounding boxes to IDs for their re-identification. The visual features are obtained using a residual neural network consisting of two convolutional layers and six residual blocks, pretrained on a person re-identification dataset with a million images [30].

BoT-SORT is a recent algorithm that achieved state-of-the-art results on multi-object tracking challenge MOT17 and MOT20 datasets [31]. It uses an appearance model based on the FastReID library [32] with features extracted using a ResNeSt50 [33] network, and Kalman filter for motion modeling. In addition, it attempts to compensate for camera motion using the global motion compensation from the OpenCV Video Stabilization module with affine transformation.

The trackers were tested on two sequences of handball practice from UNIRI-HBD dataset, with lengths of 48 s and 5 min 30 s, both at 60 frames per second, respectively. In both sequences, the camera is stationary, but in the first sequence, it zooms in at some point. The ground truth sequences were generated by detecting the players using YoloV3, “Running” the tracking using the Deep-SORT algorithm, and manually correcting the track assignments.

Since the accuracy of the object detector greatly influences the tracking accuracy, in testing, both the Deep-SORT and BoT-SORT trackers were fed the same bounding boxes generated with the Mask R-CNN detector. Since here we are mostly interested in the tracker’s abilities to assign the player detection to correct track IDs correctly. In re-identification performance, only measures that deal with track assignment are evaluated. In contrast, the commonly used Multiple Object Tracking (MOT) evaluation measures, such as Multiple Object Tracking Accuracy (MOTA) or Multiple Object Tracking Precision (MOTP) concerned with bounding box correspondences and object detection performance are ignored. Namely, the number of identity (ID) switches [34] and identification F1 (IDF1) [35] are considered.

An identity switch means that a ground truth target was assigned to a track j and the last known assignment was to a different track k ≠ j. The IDF1 focuses on how long a target is correctly identified regardless of the number of mismatches and expresses the percentage of correctly identified detections over the average number of ground truths and computed detections.

Once the players are tracked, actions annotated in time can be combined with the tracked players in a video.

#### 2.1.3. Concatenating Frames and Validation of Action Sequences

The final step involves connecting the action labels marked on a timeline with the detected player tracks. Timeline labels only contain temporal dimension and action identity but lack correspondence to the spatial location of the players performing the marked action. On the other hand, the detected player tracks locate a player in space and time in the video but lack information about the actions performed. The goal is thus to map the action labels and time ranges to corresponding player tracks, or rather, specific time ranges within a player tracking. However, since player tracking and detection are imperfect, a single-player track that should contain all appearances of that player in the video is, in fact, fragmented, so many short tracks with different IDs represent one player. Furthermore, due to missing player detections or wrong bounding boxes, the tracks may be interrupted where the player is still present on the scene or contain only part of the player that is visible in the scene. For these reasons, although an action is marked in the timeline, it is not guaranteed that a corresponding track is detected that contains the whole sequence of a player performing that action.

**Figure 3:**
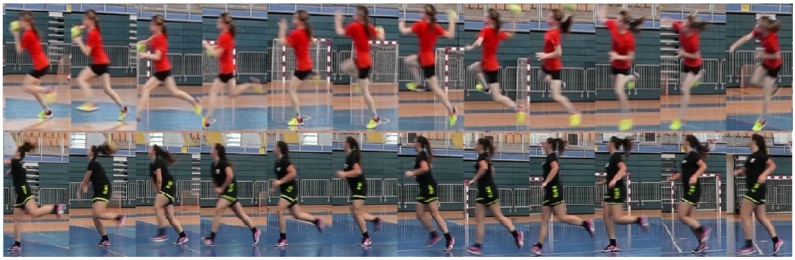
Examples of two candidates of “Jump shot” actions in an annotated time segment.

For each time segment where an action occurs, there are normally as many tracks as players in the field, and usually more due to track fragmentation (player detection is more reliable than tracking, so usually, there is at least a partial track for all players). The detected player tracks and corresponding boxes are treated as a new video sequence, where each frame is extracted from the bounding box in the original video (Figure 3). Since the dimensions of the detected bounding box may change from frame to frame to keep the frame size constant in the resulting video without resizing or changing the aspect ratio, the dimensions of the largest bounding box in the whole sequence are used for the whole sequence.

The tracks are first filtered to facilitate mapping between the temporal annotations and the detected player tracks so that only the tracks that likely contain an action are presented to the annotators. This is completed by ranking the tracks according to the optical flow (*OF*) activity measure proposed in [22]. The optical flow estimated from consecutive video frames describes the speed and direction of image patches within players bounding boxes $B_{b}$, which measures player activity [22]. The optical flow estimate is a vector field V of velocities. At each point (*x*, *y*), the vector magnitude represents the speed, and its angle represents the direction of movement. The activity measure ($A_{b}^{OF}$) of a player with its bounding box ($B_{b}$) in a single frame is calculated as the maximum optical flow magnitude $V_{x,y}$ within the area $P_{b}$ of the bounding box:

(1) $$A_{b}^{OF}=\underset{B_{b}}{\max}\left|V_{x,y}\right|:\;x,y\;\mathrm{within}\;B_{b}.$$

The activity measures for each player in individual frames are aggregated into the track scores by counting the number of times a track has the highest activity score. Finally, the top two sequences (Figure 3) were presented to the annotator according to the activity score for each temporally annotated action, to either select the sequence corresponding to the proposed action or to mark the sequence with a “Background” class if they do not contain any of the target actions or are poorly tracked.

The annotated actions are used as the ground truth in further work to create action recognition models for handball footage.

### 2.2. Action Recognition and Spatiotemporal Localization Models

A human action, according to [19], is defined as a predefined set of physical movements that a person performs in a certain time to complete an assigned task. In these physical movements, an object can sometimes be included, or more people can interact with each other to complete a more complex task.

The goal of action recognition is to assign a class label corresponding to an action (e.g., “Throw” or jump) to a video sequence. The usual assumption is that for each frame, only one pertinent label exists, i.e., only one action at a time. The recognition may be on a frame-by-frame basis, or on the level of the whole sequence, which is then also assumed to contain a single action. In the case of the unconstrained video of sports events, including handball, a more demanding task of spatiotemporal action localization is usually desired. The idea is to recognize which handball actions are performed, in which period (sequence of frames), and where the handball players performing individual actions or multiple players performing an activity are in the scene. A common approach for spatiotemporal localization, also used in this paper, is to reduce the problem to a series of action recognition tasks by generating candidate action sequences that are extracted spatially and temporally from the unconstrained video and applying the action recognition model to assign the appropriate action label. Since the generated candidate sequence does not always really show an action, the action recognition model should be able to handle that case. For that reason, in this work, we train the models on a “Background” action class which denotes a sequence that does not show a handball action or is defective due to poor player detection or tracking.

Considering the complexity and duration of certain actions and activities, some can be detected using just one frame, but for more complex actions, a sequence of frames is needed for reliable recognition, such as a “Jump Shot” consisting of several physical movements, such as “Dribbling”, jumping vertically, and simultaneously throwing a ball. For that reason, models that take the time dimension into consideration are applied to the task of handball action recognition.

**Figure 4:**
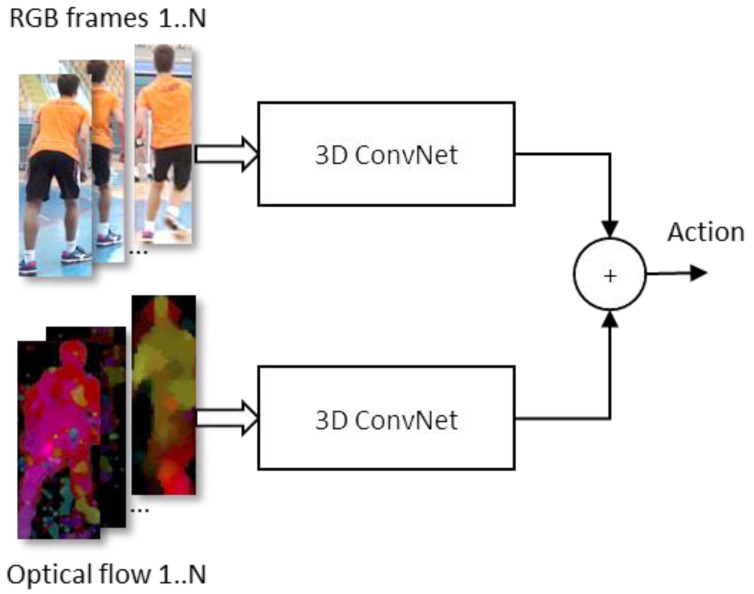
Overview of I3D model architecture.

In the following experiments, a group of models based on the Inflated 3D Network (I3D) architecture [36] were used, which was originally proposed specifically for the action recognition tasks. The I3D architecture is based on 3D convolutional neural networks that are created by “inflating” the filter and pooling layers dimensions of a 2D convolutional network (Inception-v1) into the third (temporal) dimension. The initial parameters can also be inherited from the source network by replicating the weights of the 2D filters along the time dimension. Two such inflated Inception-v1 networks are used in tandem, one to process the RGB stream consisting of unmodified video frames, and the other for processing a sequence of optical flow frames that are pre-computed from the input video (Figure 4). Both networks are independently trained on the Kinetics dataset of 400 action categories, starting from the ImageNet pretrained Inception-v1 weights. The predictions of both networks are averaged to obtain the final decision. The authors report good results with I3D architecture when fine-tuning the models pre-trained on the Kinetics dataset to fit other, smaller action recognition datasets.

In this work, the final two layers of the I3D network streams were replaced to fit the number of classes in our dataset. The models were then trained on our dataset, starting from the Kinetics pre-trained models, with all the original layers frozen and only changing the parameters of the last two layers.

The size of both RGB and optical flow frames was 224 × 224 pixels (px). In the original paper, the RGB input was prepared by first proportionally scaling the input video so that the smaller side is 256 pixels, then taking a random 224 × 224 px crop. However, in our case, since the input image is already a tight crop around a player, square cropping from a typically tall input would cut off too much of the image, such as the player’s hands, and thus lose important information about the performed action, so we opted to simply rescale the input frame to 224 × 224 px. For the optical flow stream input, the dense optical flow was first computed on the input video using Farneback’s algorithm [37], then a 224 × 224 px center crop was taken.

## 3. Action Recognition and Localization Experiments

### 3.1. Action Recognition Experiments

In the first experiment, I3D models were trained for recognition of 10 action classes (9 handball actions and “Background” classes) on UNIRI_HBD_v2 dataset.

Since the ground truth actions in the dataset contain action clips of varying lengths, during training they had to be somehow reduced or expanded to the input length of the model. In previous research [38], we investigated how different lengths of input sequences and different strategies for reduction of the number of input frames influence the action recognition accuracy and found that for some models the results improve with increasing number of input frames.

Therefore, the I3D models were trained on four different input sequence lengths, ranging from 10 to 60 frames, and using two different strategies to handle the varying sequence lengths, namely, cropping and decimation. In the cropping case, the set number of frames was extracted from the beginning part of the sequence. In the decimation case, an input sequence was reduced to the desired length by dropping frames throughout the sequence, so that the network “sees” the whole action, but at a reduced frame rate. In both cases, if the original sequence contained fewer frames than needed, the necessary number of frames was copied and inserted between existing frames to extend the sequence.

The videos were randomly divided into training and testing sets in a ratio of 80:20 for each class. The I3D models were trained using the Adam optimizer with a learning rate of 0.00005, up to 30 epochs with a batch size of 10 on an NVIDIA RTX2080 GPU. To augment the dataset, during training, in some sequences, all frames were randomly flipped left-to-right so that for example a “Shot” towards the left becomes a “Shot” towards the right side.

In the next experiment, a binary classification strategy was applied, so that for each class, a separate binary classifier was trained that decides whether a sequence should be labeled with that class or not. Each model was an I3D model as above, but with only two output classes.

All binary classifiers were trained on the same training set as above; however, the class labels were modified, so that if, e.g., a “Jump Shot” classifier was being trained, all “Jump Shot” examples were labeled as true examples, and all examples of other classes were labeled as false. In this case, there was no need for a separate classifier for the “Background” class, which was represented by the negative output of each classifier. The input sequence length was 60 frames for all classes, taken from the beginning of the original sequence, except for “Catch” and “Throw” classes, for which the input length was 20 frames.

Each model was applied to the test set and evaluated independently for its ability to classify the sequence into the correct class in terms of precision, recall, and F1 score. In this way, a sequence from the test set could be classified into zero, one, or more action classes, as it is possible that more classifiers assign their class label to the example. In the case of complex actions, such as “Dribbling” or “Passing”, this could be desirable (e.g., a sequence can simultaneously belong to the class “Catch” and be a part of class “Dribbling”). Additionally, since the input sequence length was adjusted according to individual classes, so it is shorter for “Catch” and “Throw” than for “Passing” classes, these classes can be handled better than in the multi-class model.

### 3.2. Spatiotemporal Action Localization Experiment

The most demanding task is spatiotemporal action localization, where the goal is to detect where and when actions occur in an untrimmed video and output a series of bounding box detections with the associated class labels.

**Figure 5:**
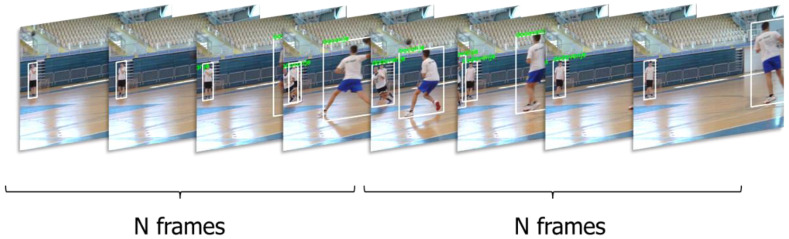
A sliding window of the candidate sequences with window length N that depends on the input length of the action recognition model and on the frame selection strategy.

For this task, we generate the candidate action sequences using the same object Mask R-CNN detector and Deep SORT tracker as in dataset preparation. The candidate sequences are then split into parts of equal duration in a sliding-window fashion (Figure 5) to be processed with an action recognition model. The window length N depends on the input length of the action recognition model and on the frame selection strategy. For the models trained using the cropping strategy, N is equal to the input length. For the decimation strategy, N can be larger than the input length and set closer to the average length of training sequences. In that case, true input to the network is again obtained by skipping frames (decimation). Finally, an action classification model is applied to each sequence to output either one of the action classes, or no action “Background” label. The same I3D models described in Section 2.1.3 are used here for this purpose.

The evaluation was performed on 6 video clips from different camera angles, of a total duration of 5 min 31 s, that were not used in training the models.

The evaluation was completed for each class separately, then the obtained results were averaged. A classified sequence was considered as true positive for a certain class if the intersection over union (IoU) overlapped between the ground truth sequence and the sequence classified into that class was at least 10% in the temporal domain. The required percentage was quite low because ground truth annotations may be much longer or shorter than the window length, e.g., a ground truth annotation of a “Throw” action in 10 frames and a window length of 60 would, in the best case, achieve an IoU of 16%. If the class is correctly assigned, but the IoU score is less than 10%, the classification is counted as a false negative. If a wrong class is assigned to a sequence, the classification is counted as a false positive for that class.

## 4. Results and Discussion

### 4.1. Object Detection Results

On the UNIRI-HBD test dataset, we compared the player detection results of the models we tuned for player and ball detection on our dataset (Yolov3, Yolov3-PB, and Mask R-CNN) with today’s improved versions of those models (Yolov7) to compare performances and to evaluate whether we need to update the data in the dataset we use to train the athlete detection model. The models were tested on a subset of 27 manually labeled images extracted from the videos of handball practice. The results of object detection in terms of average precision are shown in Table 1.

For player detection, the best results were achieved with the state-of-the-art Yolov7-e6e model, closely followed by the older Mask R-CNN model. The improvement between Mask R-CNN and Yolov7-e6e is much larger for the ball class, by nearly 15%, than for the person class, which was subjectively very good already with Mask R-CNN. Still, the ball detection performance needs to be more reliable for tracking the ball and should be still improved.

The best results for ball detection were achieved with the custom Yolov3-PB model, with an average precision of 35.44, which shows that training on custom data greatly improves the results over the basic model; however, it is still insufficient to achieve reliable ball detection. This is expected as the ball is very small and moves very fast when in the air, causing motion blur that can alter its appearance or make it virtually disappear in the “Background” and is often occluded by players. Therefore, the model comparison shows that it is unlikely that a newer model or just retraining the model on a larger amount of data will solve the ball detection problem, and it is assumed that more specific model architectures will be needed.

In the following tracking experiments, the players were detected using the Mask R-CNN detector, which was available at the time of creating the dataset, and the ball detection was not used. Since the annotation process is very time-consuming, not all videos were annotated. In the future, Yolov7-e6e or some other better-performing detector will be used for player detection in the rest of the videos.

### 4.2. Tracking Results

On the handball video from UNIRI-HBD test dataset, we compared the player tracking results achieved with Deep SORT models that were used with a fine-tuned player detectors (YOLOv3 and Mask R-CNN) with newer tracker BoT-SORT [31] that we used with newer versions of detectors (YoloV7-e6e). The aim was to evaluate whether new models can better track the player in complex scenarios and generate video sequences that better correspond to handball action to be used to train models for handball action recognition.

The reported advantage of BoT-SORT in MOT challenges [31] does not translate to our handball dataset as Deep SORT achieves slightly better IDF1 measures and produces fewer spurious tracks than BoT-SORT with either Mask R-CNN or Yolov7 used as player detectors (Table 2).

Similar with the ball case in object detection, it seems that tracking players in team sports, such as handball, differ enough from the general object tracking or tracking tuned to pedestrians, that a specialized approach is warranted that should utilize the constraints of the given sport.

**Figure 6:**
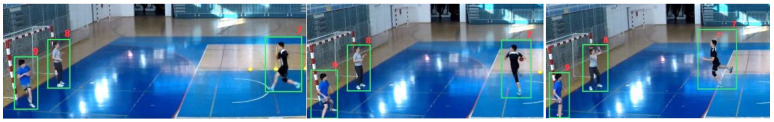
Example of tracking players with the identifications 7, 8 and 9 during three consecutive frames.

An example of parts of different chronological frames where the tracking is performed can be seen in Figure 6. The player, ID 7, is moving through the field and is successfully tracked through time.

Although both algorithms produce tracks that are much more fragmented than the ground truth, the resulting tracks are, in most cases, still long enough to contain a whole or most of a handball action.

### 4.3. Action Recognition Results

Table 3 shows action recognition results for the I3D models in terms of accuracy, precision, recall and F1 score on the UNIRI-HBD_v2 dataset. Different input length and frame selection strategies were used. The best action recognition result (F1 78%, precision 80%, recall 77%) was achieved with decimation strategy in the case of 40 frames, while the second-best result was only slightly worse (F1 77%, precision 79%, recall 76%) with the cutting strategy and 60 frames.

The F1 score of I3D_C models using the cropping strategy improves with an increasing number of frames from 10 to 60. However, the I3D_D models with the decimation strategy achieved the best result with 40 frames, slightly better than I3D_C with 60 frames.

In the case of the decimation strategy, 10 and 20 frames had the same result for an F1 score of 71%, while the increase in frames from 40 to 60 frames recorded a drop in the F1 score from 78% to 75%. Changes in precision and recall metric scores behave in a similar way, with the crop strategy they increase with the increase in the number of frames, and with the decimation they achieve the best results at 40 frames.

The sequence of 60 frames is around the average duration of video frames considering all the classes.

To analyze the results in more detail, we used the confusion matrix, which makes it easy to see whether the classifier routinely confuses two classes. Table 4 presents the confusion matrix obtained with the best preforming model I3D_40D, which uses decimation strategy and 40 input frames.

Looking at the confusion matrix, it is evident that large confusion occurs between more complex actions that include a common simpler action as its part. For example, both “Dribbling” and “Crossing” include a simpler throwing action. “Dribbling” is a complex action that involves “Running” while bouncing the ball off the floor, explaining the confusion with the “Running”, “Throw” and “Catch” classes. Similarly, “Crossing” is a complex action that consists of two “Passing” actions with a change of the players’ positions and a “Shot”, which explains the misclassifications between “Crossing” and “Passing”. The largest single confusion occurs between “Shot” and “Throw” actions, which are very similar and differ mostly by intensity and intent, a “Shot” is directed towards the goal and is usually performed with higher speed and intensity than “Throw” which signifies any other throwing of the ball, normally towards another player. “Catch” and “Throw” actions are also similar short interactions with the ball, which include a player raising his hand/hands to “Throw” or “Catch” the ball.

Considering the results achieved when observing all 10 classes together, the experiments were continued observing only one class at a time, i.e., if a certain action occurred or not.

In the second experiment, a separate I3D binary classifier was trained for each action for each sequence. The results of applying the binary classifiers on the test set are shown in Table 5, along with the results of the best-performing multi-class classifier (I3D_D with 40 input frames).

For all but one of the classes, the multi-class classifier outperformed the ensemble of binary classifiers in terms of F1 score. The results for the remaining class, “Crossing”, were rather similar for both models. Relatively problematic is the complex “Passing” action that is composed of “Catch” and “Throw” actions. The ensemble classifier would have the advantage over the multi-class classifier in this case since the ensemble classifier is not forced to choose between the “Passing” class and its parts (“Catch” and “Throw”), such as the multi-class classifier is. Instead, each binary classifier for a “Catch”, “Throw”, and “Passing” could assign its label to the “Passing” sequence. In this way, it would be expected that the ensemble classifier has overall better recall but possibly worse precision than the multi-class classifier. Surprisingly, the opposite is true here, with the ensemble classifier having a much lower recall than the multi-class classifier for both “Catch” and “Throw” classes, and the same recall with lower precision for the “Passing” class.

Additionally, the ensemble method has a large drawback in that it requires “Running” 10 models on the same example, and so requires at least 10 times the processing time than the multi-class model, and more if the time to load the models in the GPU memory is considered.

### 4.4. Action Localization Results

The averaged results of action localization are shown in Table 6.

The I3D_40D model, which achieved the best results in the action recognition task in terms of all considered metrics, also performed the best regarding the precision and F1 measures for the action localization task. In terms of recall, the I3D_40C performed slightly better at a cost of lower precision. In most cases, for the same number of input frames, the decimation models achieve better results in terms of F1 measure than the models using the crop strategy, with the only exception being the model with 60 input frames.

**Figure 7:**
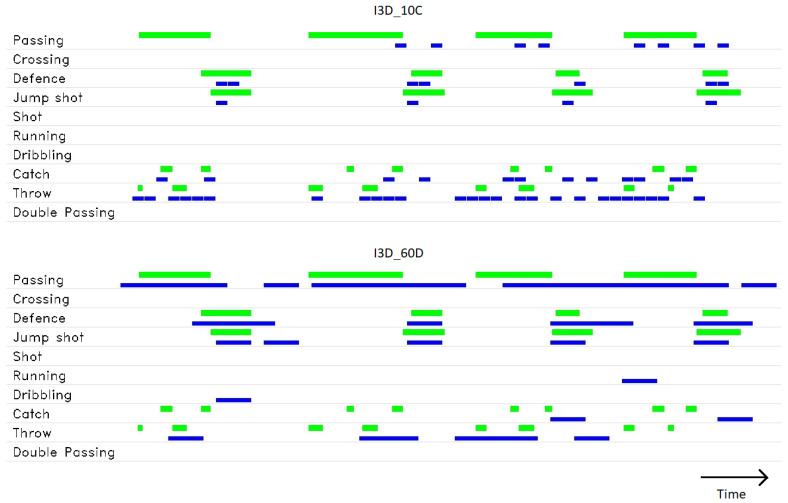
Visualization of temporal action localization timeline for I3D_10C (**top**) and 13D_60D (**bottom**). Green lines are ground truth action intervals, blue lines are detected action intervals.

An example timeline visualization of detections for a model with 10 input frames and a model with 60 input frames is shown in Figure 7. Ground truth action intervals are shown with green bars and detected action intervals with blue bars. A single ground truth action can be represented with multiple discrete detections, because of the sliding window segmentation that presents the sequence to the action recognition model in chunks of constant length. The model with fewer input frames (I3D_10C) struggles to detect longer actions, such as “Passing”, in contrast to the I3D_60D model, which takes a longer input sequence. On the other hand, the longer input limits the temporal resolution, making it difficult to detect short and quickly changing actions.

The ensemble model surprisingly did not show the best result as in the action recognition task, even with the advantage of having different input lengths for different actions.

### 4.5. Discussion

This paper considered a computer vision pipeline for analysis of activities of handball players in videos, with the final task of spatio-temporal localization of handball actions in unconstrained video. The pipeline consists of several subtasks, where object detection and player tracking provide spatially localized proposals for the sub-task of action recognition. Similar pipelines were considered for sports, such as basketball [1] and volleyball [9]. Each sub-task was considered in a separate experiment.

In the player detection task, both the Mask R-CNN and Yolov7 models already achieved good results without fine-tuning on the handball-specific dataset, with AP from about 85 to 91, depending on the configuration. However, the ball detection task was much harder, with the best AP result of 35 achieved with the Yolov3 model trained on our dataset. Training the ball detection on our dataset increased the average precision significantly, and a similar increase can be expected with newer object detection models, but the results still need to be satisfactory for real applications in handball analysis. Furthermore, since the possession of the ball performs a great role in interpreting the game situation, further specialized models for ball detection that go further than single-frame analysis should be investigated.

Generating action proposals relies on player tracking in addition to player detection. The problem of multiple object tracking, particularly tracking pedestrians, is widely researched [39]. Commonly used techniques use a combination of motion and appearance models to assist in assigning new detections to existing tracks, or in the re-identification of players in new frames. While sharing most of the challenges of pedestrian tracking, such as occlusion, bad lighting, etc., in player detection, fast and unpredictable movement makes motion prediction less reliable. Simultaneously, appearance-based re-identification is harder because different players have very similar appearances due to team jerseys. Yet, the same player can appear very different due to a much wider range of poses in sports than in pedestrians. The results of player tracking with both tested methods produce usable results for practical application in action localization; however, there is still a need for improvement. Future work should consider handball or sports specifics, such as assuming a maximum number of real identities and using more specific features, such as faces or jersey numbers for re-identification.

I3D models with different strategies (multi-class and ensemble of binary classifiers) have been investigated for the action recognition task. The best results were achieved by the multi-class model with an F1 measure of 0.78 averaged over all classes, which outperformed the ensemble of I3D models, each trained as a binary classifier for one action class with the average F1 measure of 0.69. In a future study, removing the complex actions, such as “Passing” and inferring them from their parts (“Throw” and “Catch”), will be investigated instead of direct classification of the complex actions, as these proved to be the source of most classifier confusion.

In many applications, such as video retrieval or statistics generation, it would be useful to find all actions by a certain player, which needs to be addressed in this study. However, this problem is linked to player tracking and should be considered in future work. In addition to classification accuracy, future work should focus on increasing the speed of classification, as the processing pipeline is still quite complex and computationally demanding. Approaching real-time processing would open new application possibilities in real-time statistics or highlight generation.

## 5. Conclusions

Several experiments with the application of deep learning methods for video content analysis of handball scenes were presented in this paper. Player detection, tracking, and action recognition were considered incremental steps toward the spatio-temporal localization of actions in the video, but different experiments were shown for each task.

For the action recognition task, I3D models are trained on a subset of input lengths. Next, the models are evaluated on previously extracted individual action sequences for action recognition and finally on the unconstrained video of handball training for action localization.

The set of considered actions consisted of 9 handball actions of varying complexity, from a simple “Throw” action performed by one player to complex actions, such as “Crossing” that consists of several “Throw” and “Catch” actions, and actions that involve more than one player. The varying complexity and length of actions proved challenging for action recognition tasks, where a complex action could be misclassified as one of its parts or vice versa.

For this reason, in future work, we plan to reduce the number of complex actions that are directly classified and focus on shorter and simpler actions while inferring the complex actions from the simple ones.

It would also be useful for coaches to analyze not only different actions, but also the different execution of actions by a particular player. In this sense, we plan to combine the localization of actions with the identities of players, so that coaches can guide their players to better execution of actions thanks to the possibility of filtering actions by type or player.

## Figures and Tables

**Table 1:** Object detection results on UNIRI-HBD dataset.

Model	Input Size	Ball AP	Person AP	mAP
Yolov3	608 × 608	13.53	66.13	39.83
Yolov3-PB	1024 × 1024	35.44	63.77	49.61
Mask R-CNN	1333 × 800	8.3	87.45	47.87
Yolov7 (640)	640 × 640	6.9	85.16	46.03
Yolov7-e6e	1280 × 1280	23.07	90.88	56.97

**Table 2:** Player tracking results on UNIRI-HBD dataset.

Measure	Tracker (Used Object Detector)			
	Deep SORT (Yolov3)	Deep SORT (Mask R-CNN)	BoT SORT (Mask R-CNN)	BoT-SORT (YoloV7-e6e)
Number of ground truth tracks	279			
Number of generated tracks	1554	762	1106	5646
ID switches	1483	1385	1806	1694
IDF1	24.70%	24.67%	23.26%	16.91%

**Table 3:** Action recognition results for the I3D models on the UNIRI-HBD_v2 dataset with varying input length from 10 to 60 frames and decimation and crop frame selection strategies. The best results are presented in bold.

Model	Frame Selection	Input Length	Accuracy	Precision	Recall	F1
I3D_10D	Decimation	10	0.71	0.73	0.71	0.71
I3D_10C	Crop	10	0.67	0.68	0.67	0.67
I3D_20D	Decimation	20	0.73	0.75	0.72	0.71
I3D_20C	Crop	20	0.72	0.73	0.71	0.72
I3D_40D	Decimation	40	**0.79**	**0.8**	**0.77**	**0.78**
I3D_40C	Crop	40	0.76	0.78	0.76	0.76
I3D_60D	Decimation	60	0.75	0.77	0.75	0.75
I3D_60C	Crop	*60*	*0.78*	*0.79*	*0.76*	*0.77*

**Table 4:** Confusion matrix for the I3D_40D model that uses decimation strategy and 40 input frames. The values in bold represent the percentage of samples for which the predicted label is true positive (equal to the ground-truth label).

True Class	Predicted Class									
	Passing	Crossing	Defense	Jump Shot	Shot	Running	Dribbling	Catch	Throw	Background
Passing	**82**%	1%	1%	0%	1%	2%	2%	3%	5%	3%
Crossing	13%	**79**%	3%	2%	0%	2%	2%	0%	0%	0%
Defense	2%	1%	**72**%	3%	0%	4%	2%	5%	2%	8%
Jump Shot	1%	2%	2%	**90**%	0%	4%	0%	0%	1%	0%
Shot	1%	0%	1%	11%	**56**%	6%	1%	3%	18%	1%
Running	3%	0%	1%	1%	0%	**90**%	2%	2%	1%	2%
Dribbling	7%	0%	5%	0%	0%	9%	**64**%	7%	6%	4%
Catch	6%	2%	0%	0%	0%	2%	2%	**74**%	10%	4%
Throw	7%	1%	4%	1%	2%	3%	2%	10%	**68**%	3%
Background	0%	0%	2%	0%	0%	0%	2%	1%	1%	**94**%

**Table 5:** Action recognition results for ensemble of binary I3D models and multi-class classifier I3D_D with 40 input frames on UNIRI-HBD_v2 dataset. The best average result achieved by the models on the test set is shown in bold.

Model:	Ensemble of Binary I3D Models				I3D_40D (40 Frames)		
Action Class	Input Frames	Precision	Recall	F1-Score	Precision	Recall	F1-Score
Passing	60	0.66	0.64	0.65	0.76	0.82	0.79
Crossing	60	0.9	0.74	0.81	0.88	0.79	0.74
Defense	60	0.71	0.73	0.72	0.77	0.72	0.74
Jump Shot	60	0.82	0.92	0.87	0.87	0.9	0.89
Shot	60	0.56	0.87	0.68	0.93	0.56	0.70
Running	60	0.75	0.81	0.78	0.78	0.9	0.83
Dribbling	60	0.66	0.5	0.57	0.81	0.64	0.71
Catch	20	0.73	0.53	0.61	0.71	0.74	0.73
Throw	20	0.65	0.49	0.56	0.64	0.68	0.66
**Average**		0.72	0.69	0.69	**0.80**	**0.77**	**0.78**

**Table 6:** Action localization results for I3D models with varying number and selection of frame and ensemble of I3D binary models with crop frame selection strategy. The best results of action localization are presented in bold.

Model	Frame Selection	Input Length	Window Length	Precision	Recall	F1 Measure
Ensemble	Crop	60 and 20	60 and 20	0.65	0.483	0.628
I3D_10D	Decimation	10	40	0.479	0.599	0.5
I3D_10C	Crop	10	10	0.361	0.457	0.367
I3D_20D	Decimation	20	40	0.542	0.549	0.527
I3D_20C	Crop	20	20	0.502	0.521	0.504
I3D_40D	Decimation	40	40	**0.679**	0.672	**0.646**
I3D_40C	Crop	40	40	0.606	**0.69**	0.607
I3D_60D	Decimation	60	60	0.58	0.581	0.587
I3D_60C	Crop	60	60	0.364	0.479	0.627

## Data Availability

The datasets used in this study can be found in the online repository: https://ieee-dataport.org/open-access/handball-action-dataset-uniri-hbd (accessed on 10 November 2022).
